# Demographic, clinical, and paraclinical characteristics of COVID-19 pediatric cases in southeast Iran

**DOI:** 10.1186/s13756-021-01020-8

**Published:** 2021-11-27

**Authors:** Gholamreza Soleimani, Fatemeh Akbarirad, Elham Shafighi Shahri, Seyyed Masoud Sajjadi

**Affiliations:** 1 Department of Pediatrics, School of Medicine, Children and Adolescents Health Research Center, Ali-Ibn-Abitaleb Hospital, Zahedan, Iran; 2 Department of Pediatrics, Ali-ebne-Abitaleb Hospital, Zehedan University of Medical Sciences, Zahedan, Iran; 3 Department of Pediatrics, Faculty of Medicine, Zahedan University of Medical Sciences, Zahedan, Iran; 4 Department of Cardiovascular Disease, Faculty of Medicine, Zahedan University of Medical Sciences, Zahedan, Iran

**Keywords:** COVID-19, Pediatrics, SARS-CoV-2, Clinical presentations

## Abstract

**Background:**

Even though children seem to be less vulnerable to the Coronavirus disease 2019 (COVID-19) infection, still a diverse range of clinical presentations and symptoms have been reported in children. Few studies assessed the clinical presentations of COVID-19 among Iranian children. We aimed to evaluate the clinical and paraclinical characteristics of COVID-19 infected children.

**Methods:**

All COVID-19 suspected and confirmed children were referred to the Ali-ibn-Abitaleb Hospital, Zahedan, Iran. Patients were included in this longitudinal study. Patients were evaluated at admission and during hospitalization. Patients with some of the main COVID symptoms with positive PCR test were defined as confirmed cases. Clinical, imaging and laboratory results were collected for all patients.

**Results:**

A total of 62 patients participated in this study. The male:female ratio was 1:1.03. There was a significant difference in fatigue prevalence between age groups (*P* = 0.002). There was no significant difference between groups in terms of fever duration (*P* = 0.624) and maximum temperature (*P* = 0.629). There was a significant difference between PCR positive and negative patients in terms of neurologic signs (*P* = 0.003), Intensive care unit admission (*P* = 0.001), white blood cell (*P* = 0.047).

**Conclusions:**

Even though our population was small, most of the findings matched other studies conducted on pediatric cases in Iran or other countries. It was also found that some clinical features such as pneumonia, cough, diarrhea, and tachycardia at admission time were statistically different among age groups.

## Background

Pneumonia associated with what was later called the novel coronavirus was first reported in Wuhan, China, in December 2019 [1]. The virus was formally named Severe Acute Respiratory Syndrome-Coronavirus 2 (SARS-CoV-2) due to its similarities with SARS, which emerged in 2003 [2]. Later, the disease was officially named coronavirus disease 2019 (COVID-19) by the World Health Organization (WHO). COVID-19 rapidly spread globally, and was in March 2020, WHO declared it a pandemic. Despite all efforts, the disease is still spreading and has affected people all over the world. Until July 28, 2020, more than 16 million people have contracted the virus, and around 650,000 cases lost their lives [3].

COVID-19 has been found to cause a diverse range of symptoms and complications ranging from very mild symptoms, such as sore throat and runny nose, to severe symptoms, including pneumonia, respiratory distress, and death [4–6]. In the first few months after the virus’s emergence, COVID-19 was known more as an adult disease with very few pediatric cases with mild symptoms reported in children [7–9]. For example, in the first published reports from Wuhan, no confirmed pediatric case was mentioned [10], and by January, less than 1% of COVID-19 patients in Wuhan were reported to be children [4]. A similar trend has been seen in Italy, where till July 21, 2020, 1% and 1.7% of the confirmed cases were children, respectively in the age range of 0–9 and 10–19 years old, and 4 cases of death were reported among children [11]. Similarly, in December 2020, 10.2% of confirmed COVID-19 cases in the United States were children [12].

However, later and especially after March 2020, some children infected with COVID-19 presented with severe clinical symptoms similar to Kawasaki disease’s (KD) diagnosis. The condition was termed multisystem inflammatory syndrome in children (MIS-C) [13]. Further research showed that similar to adults, the clinical presentations of COVID-19 in children were not consistent and might include a diverse range [14, 15].

Iran was one of the first countries badly hit by the virus. Although, as of July 2020, the highest incidence rate of COVID-19 infection in the Middle East was reported in Qatar (38939.1 per million population), the cause-specific mortality in Iran was the highest in the region (212.5 per million population) [16]. Unfortunately, despite all efforts, Iran had 610 confirmed new cases per million population and reported more than 53,000 deaths from the beginning of the pandemic till December 20, 2020 [3]. There have been some studies on the epidemiological analysis of COVID-19 in the Iranian population [17, 18], but to the best of our knowledge, very few comprehensive studies have been conducted on this disease’s clinical presentations among children [19, 20]. Therefore, the present research was aimed to evaluate clinical and paraclinical characteristics of COVID-19 infected children in Zahedan city, southeast of Iran, to provide insights on the early diagnosis and efficient management of COVID-19 infection in children.

## Methods

### Study design and population

All pediatric patients (under the age of 18 years old) admitted due to suspicion of COVID-19 infection, with likely COVID-19 infection or confirmed diagnosis of COVID-19 between March 21 and November 30, 2020, were recruited in the survey. The study was conducted in Ali-ibn-Abitaleb Hospital of Southeast Iran, affiliated with the Zahedan University of Medical Sciences.

A suspected case was defined as a patient with a dry cough, chills, or sore throat with shortness of breath, with or without fever, with no other known etiology for the symptoms. Besides, a patient with fever and shortness of breath, diarrhea, nausea and vomiting, headache, abdominal pain, with or without Kawasaki disease (KD)-like symptoms who had been in close contact with a suspected or confirmed case of COVID-19 in the previous 14 days was also considered as a suspected case.

A likely cause was defined as a patient having imaging findings corresponding to COVID-19. Additionally, patients with pneumonia who did not respond to treatments and whose clinical condition deteriorated rapidly or resulted in death were considered likely cases. Furthermore, patients whose polymerase chain reaction (PCR) test was unclear and inconclusive (neither positive nor negative) were also considered likely. A real-time PCR method was used to confirm the COVID-19 cases. For this purpose, COVID-19 onestep RT-PCR kit (Pishtaz teb, Iran) was used. The kit was designed to detect dual target genes of COVID-19 nycleocapsid (*N* gene) and RNA dependent RNA polymerase (*RdRp* gene). *RNAseP* gene was used as an internal control. All runs were performed using the RT-PCR device (Rotor gene Q, QIAGEN, Germany) according to the kit instructions [21].

Finally, a patient with some of the symptoms discussed above whose PCR test was positive was defined as a confirmed case.

### Inclusion and exclusion criteria

Inclusion criteria were children (0–18 years) admitted from March 21 to July 21, 2020, as suspected, likely, or confirmed case of COVID-19. Patients with a confirmed diagnosis other than COVID-19 during admission were excluded.

### Study procedure

At the admission time, routine laboratory tests, chest X-ray imaging, and a computerized chest tomography (CT) scan were performed. Chest CT-scan was performed using CT-Scan device (16 Slides, Toshiba, Japan) by an expert operator. All findings along with clinical features of the patients were recorded. Accordingly, a questionnaire was completed by the care team with the participation of the patient and their parents. The questionnaire included demographic information and questions about potential exposure and source of transmission, and whether anyone else in the family had been a confirmed or suspected case of COVID-19.

### Statistical analyses

The statistical package for social sciences (SPSS) software version 23.0 was used for statistical analysis. The frequency distribution of features among age groups was compared using the chi-square or Monte Carlo tests. Comparison of the continuous variables between groups was performed using the Kruskal–Wallis test due to the non-normal distribution of the variables based on the Shapiro–Wilk test. A *P* value smaller than 0.05 was considered statistically significant.

## Results

**Fig. 1:**
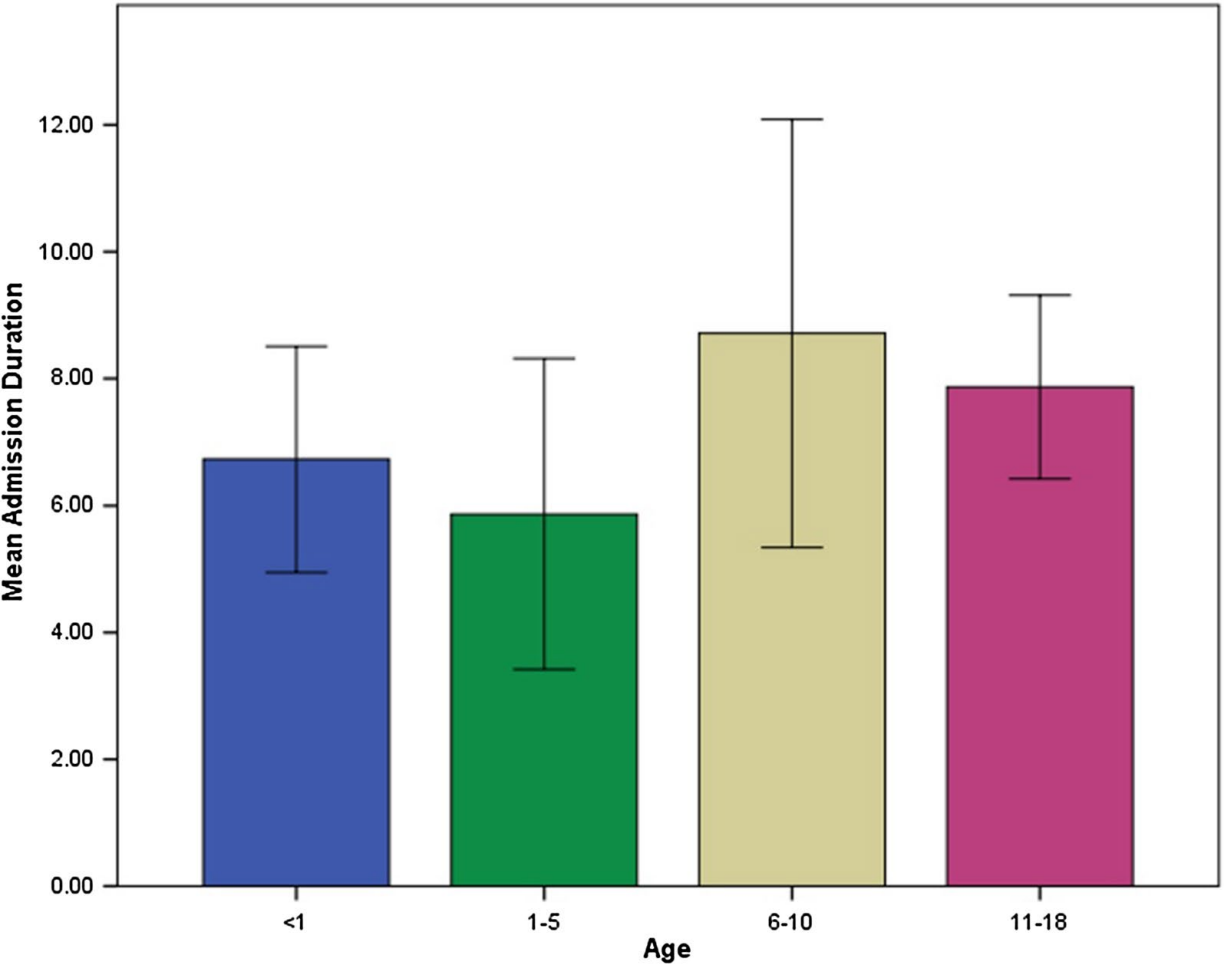
Admission duration among age groups

There were no death cases through the study. Sixty-two pediatric patients, < 1–18 years old, were included in the study. Of them, 49 (79.0%) were confirmed cases with a positive PCR test. The patients were divided into four groups of age as follows: < 1 year (n = 11, 17.7%), 1–5 years (15, 24.2%), 6–10 years (21, 33.9%), and 11–18 years (15, 24.2%). The male:female ratio was 1:1.03. The clinical and paraclinical findings of the patients are summarized in Table 1. Laboratory findings and the duration of hospitalization (quantitative features) are presented in Table 2. There was a significant difference in fatigue prevalence between age groups (*P* = 0.002). Fatigue was common among the 6–10-year-old group. The median and interquartile range (IQR) for the patients’ admission duration was 7.00 and 5.25 days, respectively. There was no significant difference in admission duration between age groups (*P* = 0.231) (Fig. 1).

**Table.1:** Demographic, clinical, and imaging features of the patients

Variable	Categories/feature	Positive cases					*P* value
		Total N (%)	< 1 n (%)	1–5 n (%)	6–10 n (%)	11–18 n (%)	
Age (years)	< 1	8 (72.7%)	–	–	–	–	–
	1–5	15 (100.0%)	–	–	–	–	
	6–10	15 (71.4%)	–	–	–	–	
	11–18	11 (73.3%)	–	–	–	–	
Exposure	Confirmed family member	10 (20.4%)	1 (12.5%)	4 (26.7%)	3 (20.0%)	2 (18.2%)	0.677^ǂ^
	Suspect family member	14 (28.6%)	3 (37.5%)	4 (26.7%)	4 (26.7%)	3 (27.3%)	
	Unknown source	20 (40.8%)	2 (25.0%)	7 (46.7%)	7 (46.7 %)	4 (36.4%)	
	Contact with other suspects	3 (6.1%)	2 (25.0%)	0 (0.0%)	0 (0.0%)	1 (9.1%)	
	Not-mentioned	2 (4.1%)	0 (0.0%)	0 (0.0%)	1 (6.7%)	1 (9.1%)	
Diagnosis	Asymptotic infection	11 (22.4%)	4 (50.0%)	3 (20.0%)	1 (6.7%)	3 (27.3%)	0.097^ǂ^
	URTI	14 (28.6%)	1 (12.5%)	6 (40.0%)	4 (26.7 %)	3 (27.3%)	0.629^ǂ^
	Pneumonia	37 (75.5%)	5 (62.5%)	13 (86.7%)	13 (86.7%)	6 (54.5%)	0.194^ǂ^
Clinical findings	Fever	47 (95.9%)	8 (100.0%)	14 (93.3%)	15 (100.0%)	10 (90.9%)	0.839^ǂ^
	Cough	31 (63.3%)	4 (50.0%)	12 (80.0%)	10 (66.7%)	5 (45.5%)	0.263^†^
	Tachypnea^a^	29 (59.2%)	5 (62.5%)	7 (46.7%)	11 (73.3%)	6 (54.5%)	0.503^†^
	Blood oxygen saturation < 92 %	27 (55.1%)	6 (75.0%)	5 (33.3%)	9 (60.0%)	7 (63.6%)	0.202^†^
	Tachycardia^a^	25 (51.0%)	6 (75.0%)	5 (33.3%)	8 (53.3%)	6 (54.5%)	0.283^†^
	Pharyngeal erythema	24 (49.0%)	2 (25.0%)	9 (60.0%)	6 (40.0%)	7 (63.6%)	0.262^†^
	Fatigue	20 (40.8%)	0 (0.0%)	3 (20.0%)	10 (66.7%)	7 (63.6%)	0.002^†^*
	Vomiting	20 (40.8%)	3 (37.5%)	4 (26.7%)	7 (46.7%)	6 (54.5%)	0.503^†^
	Diarrhea	16 (32.7%)	4 (50.0%)	3 (20.0%)	4 (26.7%)	5 (45.5%)	0.355^†^
	Nasal congestion	15 (30.6%)	3 (37.5%)	3 (20.0%)	5 (33.3%)	4 (36.4%)	0.742^ǂ^
	Runny nose	15 (30.6%)	1 (12.5%)	6 (40.0%)	4 (26.7%)	4 (36.4%)	0.548^ǂ^
Chest X-ray	Bilateral haziness	20 940.8%)	2 (25.0%)	6 (40.0%)	7 (46.7%)	5 (45.5%)	0.767^†^
	Peripheral opacity	15 (30.6%)	2 (25.0%)	5 (33.3%)	5 (33.3%)	3 (27.3%)	0.964^†^
Chest CT scan	Ground-glass opacity	29 (59.2%)	3 (37.5%)	12 (80.0%)	9 (60.0%)	5 (45.5%)	0.164^†^
	Local patchy shadowing	5 (10.2%)	1 (12.5%)	0 (0.0%)	2 (13.3%)	2 (18.2%)	0.565^ǂ^
	Bilateral patchy shadowing	11 (22.4%)	2 (25.0%)	2 (13.3%)	3 (20.0%)	4 (36.4%)	0.726^ǂ^
	Interstitial abnormalities	11 (22.4%)	2 (25.0%)	4 (26.7%)	3 (20.0%)	2 (18.2%)	0.999^ǂ^
Neurological signs	Reduced consciousness	10 (20.4%)	2 (25.0%)	1 (6.7%)	5 (33.3%)	2 (18.2%)	0.403^ǂ^
	Seizure	9 (18.4%)	4 (50.0%)	2 (13.3%)	2 (13.3%)	1 (9.1%)	0.129^ǂ^
	Total symptoms	21 (42.9%)	4 (50.0%)	12 (80.0%)	5 (33.3%)	7 (63.6%)	0.097^ǂ^
Liver involvement		15 (30.6%)	2 (25.0%)	6 (40.0%)	4 (26.7%)	3 (27.3%)	0.823^†^

*Significant difference

^ǂ^The Monte Carlo test was used for the comparison

^†^The chi-square test was used for the comparison

^a^Measured at hospital admission

**Table.2:** Association between PCR result and study parameters

Variables	PCR positive N (%)	PCR negative N (%)	*P* value
Neurologic signs	21 (42.9%)	0 (0.0%)	0.003*^†^
Liver involvement (> 4 fold increase in ALT or AST)	15 (30.6%)	2 (15.4%)	0.485^†^
Lymphopenia at admission (lymphocyte count < 1000/ml)	10 (20.4%)	5 (38.5%)	0.177^†^
Pulmonary involvement in imaging	49 (100.0%)	13 (100.0%)	–
ICU admission	24 (49.0%)	0 (0.0%)	0.001*^†^
Fever	47 (95.9%)	12 (92.3%)	0.513^†^

Variables	PCR positive mean (SD)	PCR negative mean (SD)	*P*
WBC (/ml)	12170.00 ± 7337.02	8877.78 ± 5266.35	0.047*^‡^
Seg (%)	68.09 ± 18.97	73.56 ± 14.47	0.815^‡^
Lymph (%)	19.75 (26.00)	15.00 (19.00)	0.736^ǂ^
PLT (1000/ml)	289040.00 ± 221598.00	258777.78 ± 151754.55	0.141^‡^
Hb (mg/dl)	10.10 (2.80)	12.00 (3.30)	0.494^ǂ^
BUN (mg/dl)	10.00 (7.00)	12.00 (6.00)	0.835^ǂ^
Cr (mg/dl)	0.55 (0.27)	0.60 (0.40)	0.269^ǂ^
ALT (mg/dl)	71.50 (131.00)	44.00 (39.00)	0.009^ǂ^
AST (mg/dl)	54.00 (94.00)	52.00 (130.00)	0.059^ǂ^
LDH (mg/dl)	610.00 (485.00)	853.89 ± 444.56	0.194^ǂ^
CPK (mg/dl)	130.00 (154.00)	329.89 ± 331.73	0.332^ǂ^
ESR	32.50 (56.00)	48.89 ± 26.34	0.665^ǂ^
CRP	96.00 (72.00)	96.00 (64.00)	0.271^ǂ^
PT	13.00 (2.00)	14.44 ± 2.26	0.394^ǂ^
PTT	31.00 (5.00)	34.00 (9.00)	0.404^ǂ^
INR	1.10 (0.20)	1.20 (0.30)	0.308^ǂ^

*Significant difference

^†^Comparison was performed using Fisher exact test

^‡^Comparison was performed using an independent t-test

^ǂ^Comparison was performed using the Mann–Whitney test

**Fig. 2:**
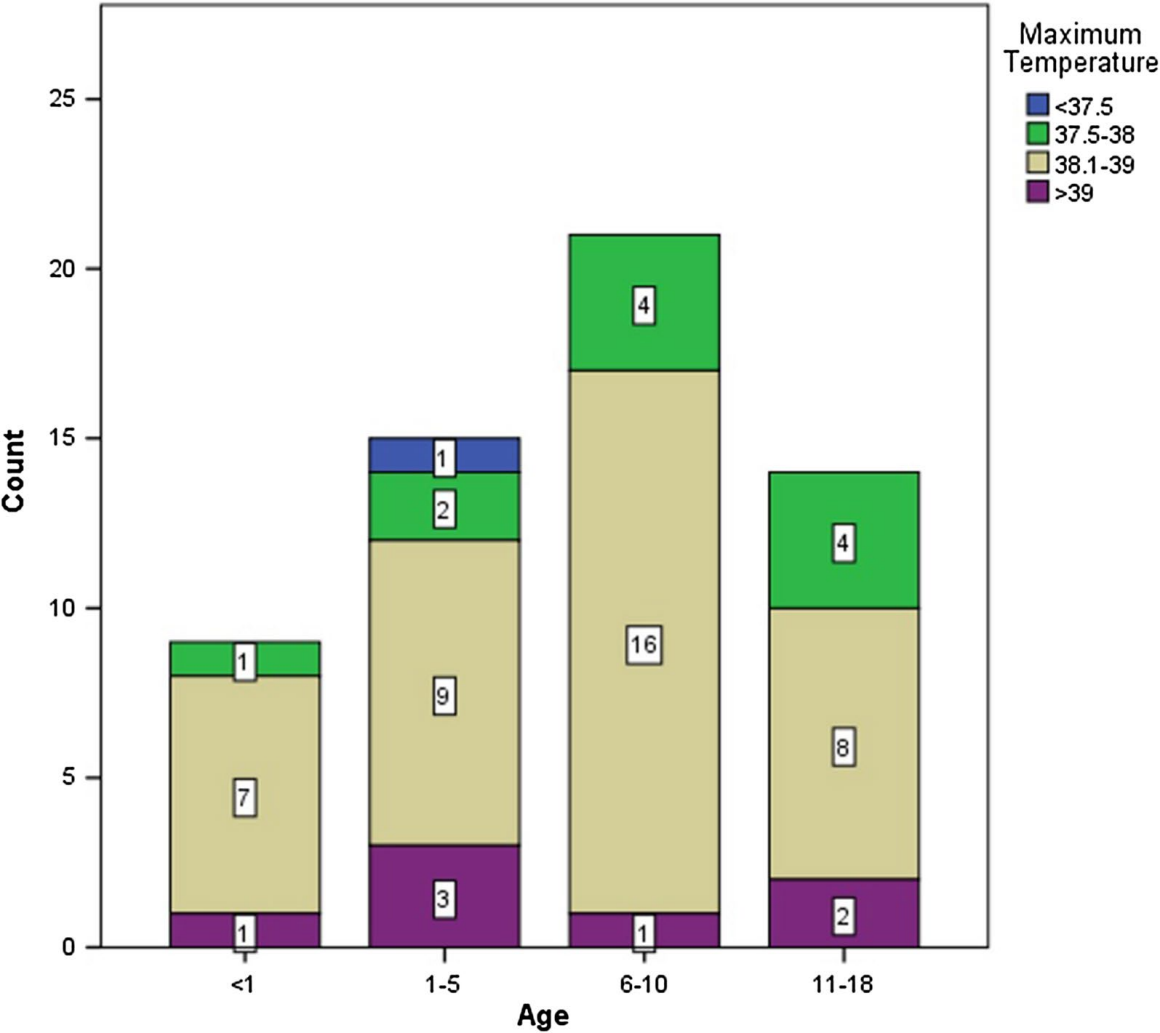
Distribution of maximum temperature among age groups

The median and IQR for fever duration were 3.50 (3.00) and 5.00 (3.00) in the age group below 1-year-old and 1–5 years old groups. The mean and standard deviation (SD) for fever duration was 5.21 ± 1.96 and 5.50 ± 2.24 in 6–10-year-old and 11–18-year-old groups. There was no significant difference between groups in terms of fever duration (*P* = 0.624). The maximum temperature distribution among age groups is shown in Fig. 2. There was no significant difference in the distribution of maximum temperature between age groups (*P* = 0.629).

Meningoencephalitis was observed in one patient (1–5-year-old group), transverse myelitis with lower-limb paralysis and sensory disorder were observed in one patient (6-10-year-old group).

The association between study parameters and PCR positivity is presented in Table 2. There was a significant difference between PCR positive and negative patients in terms of neurologic signs (*P* = 0.003), intensive care unit (ICU) admission (*P* = 0.001), white blood cell (WBC) (*P* = 0.047).

## Discussion

In this study, 62 COVID-19 pediatric patients (suspected, likely, and confirmed) were evaluated, and their clinical and paraclinical features, including symptoms at admission, imaging findings, and laboratory analysis results along with the demographic information and potential source of exposure, were analyzed.

In our study, the sex distribution was almost balanced. Previous studies also showed no substantial difference between genders, 51% boys in Italy until July 21, 2020 [11], 57% in the United States [21], and 56.6% boys in China [9]. In another study on Iranian children by Soltani et al. [19], 46.7% of the evaluated 30 children were male [19].

Our study indicated that fever was the most common symptom (95.9% of cases) followed by cough (63.3%), tachypnea (59.2%), and reduced blood oxygen saturation (55.1%). In the study by Lu et al. on 171 confirmed pediatric cases of COVID-19 in Wuhan, cough (48.5%), erythema (46.2%), and fever (41.5%) were the most frequent symptoms [8]. However, fever was much more frequent among our cases. Besides, 75.8% of our patients had a fever of > 38 °C compared to only 32.1% in the study of Lu et al. [8]. In the Centers for Disease Control and Prevention (CDC) report, fever (56%) and cough (54%) were the most common symptoms among 291 American pediatric patients, which was in line with the findings of our study [22]. Our results were also in line with a systematic review that reported fever in 60.0–76.1% of pediatric patients based on a case series and a cross-sectional study in China [15]. In a study on Iranian children, fever was reported in 76.7% of patients, followed by tachypnea (76.7%), dyspnea (66.7%), and cough (53.3%), which were consistent with our results [19]. The same three symptoms and signs of fever, cough, and tachypnea were reported in all 9 Iranian patients in a case series by Rahimzadeh et al. [20].

One of the potential reasons for the difference in fever frequency in different studies can be reporting the fever at admission time versus during hospitalization. In most studies, regardless of the subject’s age, fever was usually the most common symptom ranging from 71 to 98.6% of subjects [4, 6, 18, 22].

Considering the least frequent symptoms, our findings correspond to most other studies which reported diarrhea and vomiting as the least common ones. However, while in our study, diarrhea, nasal congestion, and runny nose were found in 32.7%, 30.6%, and 30.6%, respectively. Diarrhea and vomiting were previously reported to be the least common symptoms in children with COVID-19 infection [8, 19, 22]. The difference between the other findings can probably be due to the small size of our study.

The median admission duration was 7 days in our study. Previous studies reported the median admission duration ranged from 3 to 6 days [23, 24]. Although our study’s sample size was small, it should be noted that some previous reports aggregated data mostly from walk-in COVID-19 test centers [22], while our study was conducted on patients who were referred to the hospital, which usually occurs when the symptoms are more severe.

The potential source of exposure was unknown in 40.8% of our study while having a suspected or confirmed family member was recorded in 28.6% of the patients. A previous study reported that having a suspected or confirmed family member accounted for 90.1% of the patients, while the source was unknown only in 8.8% [8]. In the Center of Diseases Control (CDC) report, the exposure information was only available for 184 out of 2572 pediatric patients (7.2%), the most common source of exposure was exposure in the family [22]. In a study on Persian children, the source of exposure was reported only for 36.7% of the cases, having close contact with a suspected or confirmed case of COVID-19 [19]. Expression of information about the source of exposure is challenging. Furthermore, our study’s sample size and the social conditions, including school closure strategies, travel ban, and business luck downs, differ between societies. These factors can affect the frequency distribution of potential sources of exposure. In our study there was no significant difference in source of exposure between age groups.

Regarding the imaging findings, CT scan revealed ground-glass opacity in 59.2% of cases, which was in line with the findings of previous studies that reported ground glass appearance in 32.7–73.1% of the patients [8, 19, 25]. Local and bilateral patchy shadowing was not frequent in our study (10.2% and 22.4%, respectively). A similar finding was reported in previous studies on children (18.7% and 12.3%) [8] and patients regardless of age (ground-glass opacity in 56.4%, patchy bilateral shadowing in 51.8%) [4].

Our study findings showed that neurological symptoms were seen in 42.9% of the patients. The prevalence of neurologic signs was significantly higher in patients with positive PCR than those with negative PCR (42.9% and 0.0%, respectively). The prevalence of neurological symptoms was higher in our study than the previously reported prevalence of 14.8–22% [24, 26]. The difference between the study findings might be due to the difference in the studies’ sample size. Our study’s findings showed that the most common neurological symptom was reduced consciousness (20.4%) followed by seizure (18.4%). Reduced consciousness was more prevalent in the 6–10-year-old group, but the difference was not statistically significant. The seizure was more common among children below 1 year of age in our study. Similar to our study’s findings, reduced consciousness was most common among older children in a previous study, while hypotonia was the predominant neurological sign in children under 2 years of age [24]. In another study, the most common neurological symptom was encephalopathy followed by headache and brain stem signs; these findings were not compatible with our study’s pattern of neurological symptoms [26]. The difference might be due to the difference in age groups and sample size of the studies.

In our study, 24 (38.7%) patients were admitted to ICU. Intensive care unit admission was significantly more common among PCR positive patients than PCR negative patients (49.0% and 0.0%, respectively). The ICU admission among children with COVID-19 infection was previously reported to range from 0.1 to 24% [24, 27, 28]. More extensive studies revealed a lower prevalence of ICU admission. Therefore, our study’s findings will fit in a small study category and therefore might not be attributed to the whole population of COVID-19 infected children.

Our study also found that WBC and platelet count was elevated in study patients. However, the WBC count was significantly higher in patients with negative PCR (8877.78 ± 5266.35) than the positive PCR test (12170.00 ± 7337.02). Elevated WBC and platelet count were also reported in previous studies of COVID-19 infected patients [29, 30].

Our study findings also showed that Erythrocyte Sedimentation Rate (ESR) and C-Reactive Protein (CRP) were high in both PCR positive and negative patients. However, high CRP and ESR levels should be associated with the COVID-19 infection, as reported in other studies. In the study by Cai et al. [23] on 10 Chinese children, 6 had high CRP levels even though the maximum CRP level was 35 mg/l [31]. In the study by Soltani et al. on 30 Iranian children, positive CRP was reported in 23 out of 30 cases (77%), and abnormal ESR was seen in 11 out of 25 cases (44%), which matched our findings [19]. Besides, 100% and 90% of 10 patients in the study by Rahimzadeh et al. had positive CRP and elevated ESR, respectively [20].

In a retrospective study on 2143 Chinese children, they concluded that children of all ages are susceptible to be infected with COVID-19, and even though their clinical manifestations are generally milder, younger children, especially infants, are at risk [9]. The mechanism of action of SARS-CoV-2 has become more precise thanks to its resemblance to SARS [32]. However, the reason for children being less prone to COVID-19, what Cristiani et al. call the “secret” of children, is not still evident. They concluded that it should be due to the interaction between the children’s immunological response and the virus pathogenetic mechanisms [7]. They discussed that the expression of angiotensin-converting enzyme 2 (ACE2) and lymphocyte count is higher in children compared to adults. Since children’s immune system experiences frequent viral infections, children’s immune system is more trained and adaptive than adults, and therefore children experience milder symptoms compared to adults [7].

The main limitation of this study was the single-center study design. A single-center study resulted in obtaining a small number of patients. However, targeting < 1–18 years old pediatric patients and specific detection of COVID-cases are important strenght points of the survey.

## Conclusion

Children at any age seem to be susceptible to COVID-19, and even though their symptoms are milder, they still present a diverse range of clinical presentations. This study reported demographic, clinical, imaging, and laboratory findings of 62 children as suspected, likely, or confirmed cases of COVID-19 in Zahedan, Iran. Even though our population was small, most of the findings matched other studies. It was also found that only fatigue was statistically different among age groups. In terms of laboratory findings, CRP, ESR, lymphocyte, platelets, and WBC were high. While we hope that no larger patients will be seen, further studies may be conducted by aggregating data from different centers and regions to have more conclusive insights.

## Data Availability

All data generated or analyzed throughout this research are included in this published article.

## Abbreviations

COVID-19: Coronavirus disease 2019
SARS-CoV-2: Severe acute respiratory syndrome-Coronavirus 2
WHO: World Health Organization
KD: Kawasaki Disease.
MIS-C: Multisystem inflammatory syndrome in children
PCR: Polymerase chain reaction
CT: Chest tomography
SPSS: Statistical package for social sciences
IQR: Interquartile range
SD: Standard deviation
ICU: Intensive care unit
WBC: White blood cell
ESR: Erythrocyte sedimentation rate
CRP: C-reactive protein
